# Bilateral Numb Chin Syndrome as the Initial Presentation of Burkitt's Lymphoma/Leukemia: A Report of Two Cases and Review of the Literature

**DOI:** 10.1155/2016/3791045

**Published:** 2016-11-01

**Authors:** Hussein Algahtani, Bader Shirah, Wafaa Bassuni, Reem Adas

**Affiliations:** ^1^King Abdulaziz Medical City, King Saud bin Abdulaziz University for Health Sciences, Jeddah, Saudi Arabia; ^2^King Abdullah International Medical Research Center, King Saud bin Abdulaziz University for Health Sciences, Jeddah, Saudi Arabia

## Abstract

Numb chin syndrome, also known as mental nerve neuropathy, is a rare sensory neuropathy characterized by paresthesia and hypoesthesia in the area supplied by the mental nerve and its branches. This syndrome may be the first symptom of underlying malignancy or the first sign of recurrence and metastasis in patients with preexisting cancer. In this article, we present two cases with bilateral numb chin syndrome as the first manifestation of Burkitt's lymphoma/leukemia and review the relevant literature. Numb chin syndrome should be considered as a warning sign and raise the suspicion for an underlying malignancy. Bilateral involvement is especially hazardous and must not be underestimated. In fact, an astute neurologist and internist who realizes that chin numbness is a potentially significant symptom can then exclude serious underlying malignancies. Standard diagnostic protocol with different modalities of imaging based on the availability and experience of the radiology team should be mandatory. High index of suspicion should be practiced to avoid delay in diagnosis and progression of the underlying malignancy.

## 1. Introduction

Numb chin syndrome, also known as mental nerve neuropathy, is a rare sensory neuropathy characterized by paresthesia and hypoesthesia in the area supplied by the mental nerve and its branches. This syndrome may be the first symptom of underlying malignancy or the first sign of recurrence and metastasis in patients with preexisting cancer [1]. It has been reported in association with several hematologic malignancies, thyroid, prostate, and breast cancer. It usually presents unilaterally, but bilateral manifestations have also been reported [2]. The pathophysiologic mechanism of this syndrome is either infiltration or compression of the inferior alveolar nerve or the mental nerve. It is associated with poor prognosis especially in patients with metastatic disease. The presence of numb chin syndrome should warrant careful examination, investigation, and monitoring of the affected patients [3]. In this article, we present two cases of numb chin syndrome as the first manifestation of Burkitt's lymphoma/leukemia and review the relevant literature.

## 2. Case Report 1

**Figure 1:**
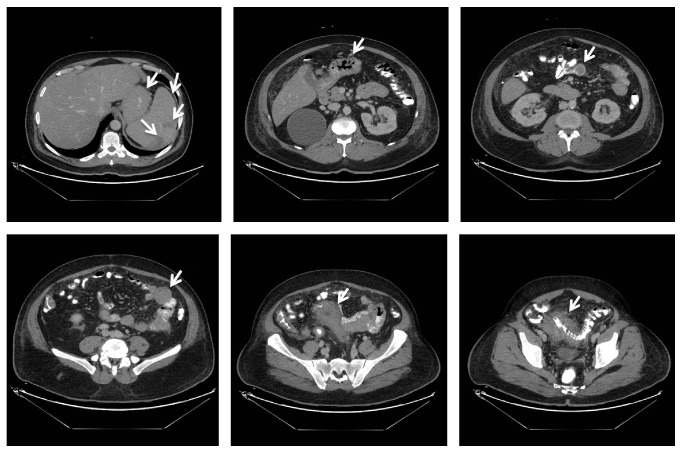
CT NCAP demonstrating a large soft tissue mass surrounding the sigmoid with mild enhancement measuring around 9.2 × 12 × 6 cm, multiple enhanced soft tissue masses surrounding multiple segments of the small bowel, multiple peritoneal implants, and multiple mesenteric subcentimetric lymph node enlargement. There were also circumferential stomach wall thickening measuring around 2.8 cm, small subcentimetric left axillary and periaortic lymph nodes, splenomegaly, and two splenic enhancing lesions.

**Figure 2:**
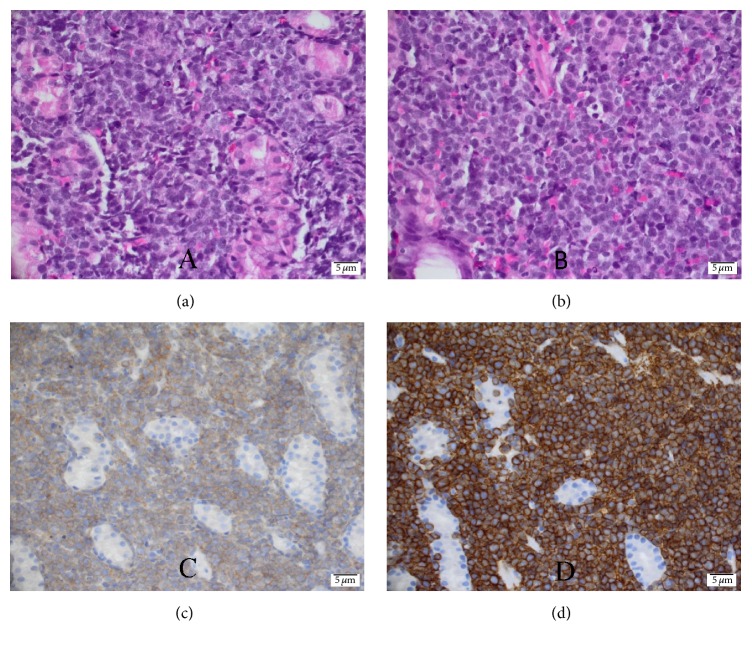
Histopathology of Burkitt's lymphoma cells from duodenal biopsy showing moderately enlarged hyperchromatic nuclei with no prominent nucleoli and starry sky appearance under the microscope with hematoxylin and eosin stain (a & b), CD10 positivity (c), and CD 20 positivity (d).

**Figure 3:**
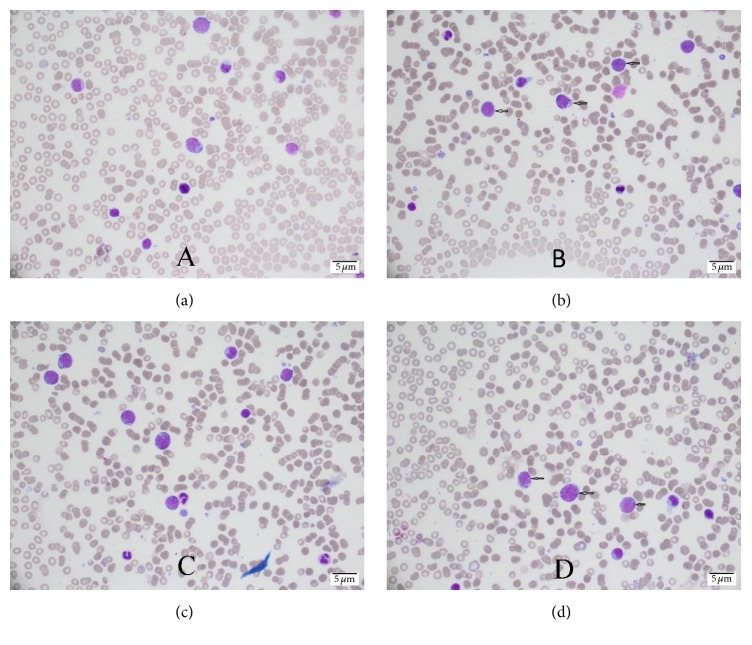
Bone marrow aspiration biopsy showing extensive (40%) infiltration by mononuclear cells with high nuclear : cytoplasmic ratio, basophilic cytoplasm, and cytoplasmic vacuolation (Burkitt's lymphoma cells).

**Figure 4:**
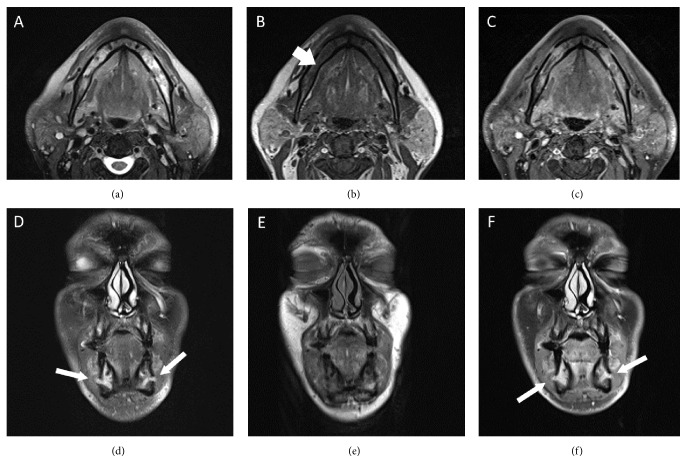
Axial MRI images at the level of the mandible demonstrating abnormal bone marrow infiltrative process replacing the normal bone signal on both T2-weighted images (a) and T1-weighted images (b) with diffuse enhancement on the contrast-enhanced axial T1-weighted images (c) (short arrows). There were no pathologic fractures and no soft tissue masses. Coronal T2-weighted images (d), unenhanced T1-weighted images (e), and contrast-enhanced T1-weighted images (f), showing abnormal thickening and enhancement of the mental nerves as they exit the mental foramen bilaterally (long arrows).

A 57-year-old male patient, known case of diabetes mellitus type 2 for 2 years and hypertension for 7 years, presented with bilateral numbness in the lower lip and chin for one-week duration. The numbness was continuous with no associated pain, weakness, or any other neurological symptoms. There was no history of recent trauma or dental extraction. On initial presentation, there were no signs of fever, anemia, fatigue, headache, or lymphadenopathy. There was no mandibular swelling, gingival abnormalities, or skin rash. Neurological examination was normal apart from hypoesthesia to thermal stimulation and pinprick over the distribution of mental nerve bilaterally. Initial peripheral full blood examination was normal. The patient was advised to undergo a thorough medical examination to look for underlying malignancy including magnetic resonance imaging (MRI) of the face and brain, computed tomography (CT) Neck/Chest/Abdomen/Pelvis (NCAP), and tumor marker, but unfortunately he elected to leave for a business trip. Two weeks later, the patient developed fever, night sweats, and fatigue. One week later, the patient's condition deteriorated, and he developed pneumonia, which necessitated an immediate return to Saudi Arabia and admission to our hospital for detailed workup. Social history was significant for smoking hookah for 30 years. His family history was unremarkable. Examination on admission showed an ill-looking male with fever, pallor, shortness of breath, and fatigue. Neurological examination redemonstrated light touch and pinprick sensation in the distribution of the mental nerve bilaterally with no other neurological deficits. CT NCAP demonstrated a large soft tissue mass surrounding the sigmoid with mild enhancement measuring around 9.2 × 12 × 6 cm. In addition, there were multiple enhanced soft tissue masses surrounding multiple segments of the small bowel, multiple peritoneal implants, and multiple mesenteric subcentimetric lymph node enlargement. There was circumferential stomach wall thickening measuring around 2.8 cm. Other abnormalities included small subcentimetric left axillary and periaortic lymph nodes, splenomegaly, and two splenic enhancing lesions (Figure 1). Upper gastrointestinal endoscopy with specimens from the stomach and duodenum revealed fragments of mucosal tissue with prominent lamina propria expansion and monomorphic lymphoid infiltration. The lymphocytes had moderately enlarged hyperchromatic nuclei with no prominent nucleoli. Numerous mitoses and focal necrosis were present. Neoplastic cells were positive for CD10, CD20, CD45, BCL-6, CD43, and Ki-67. These cells were negative for CD3, CD5, CD30, and BCL-2 (Figure 2). Flow cytometric analysis of tissue biopsy showed mature T cells with a CD4/CD8 ratio of 0.34 and around 67% B-cells expressing CD10, CD19, CD20, and CD45 and showed surface immunoglobulin kappa light chain restriction. MRI of the brain was normal, but MRI of the face showed bilateral abnormal enhancement of the mental nerve at the level of the mental foramen, most likely related to leukemic infiltration (Figure 3). Bone marrow aspiration biopsy demonstrated extensive (40%) infiltration by mononuclear cells of Burkitt's type (Figure 4). The finding was consistent with B-cell lymphoproliferative disease of Burkitt's type (Burkitt's lymphoma/leukemia). His blood workup showed elevated lactate dehydrogenase level, creatinine, and uric acid. Peripheral blood film showed around 18% B-cells expressing CD10, CD19, HLADR, and CD45 and negative for CD7 and CD34. His cerebrospinal fluid (CFS) analysis showed a clear fluid with no white blood cells (WBCs), red blood cells (RBCs), or blast cells and normal biochemical profile. The final diagnosis was Burkitt's lymphoma/leukemia. The patient was commenced on antibiotics, allopurinol, and diuretics. His condition improved transiently which allowed the initiation of chemotherapy cyclophosphamide, doxorubicin, vincristine, and prednisolone. The patient's condition improved, and currently he is completing his chemotherapy cycles.

## 3. Case Report 2

**Figure 5:**
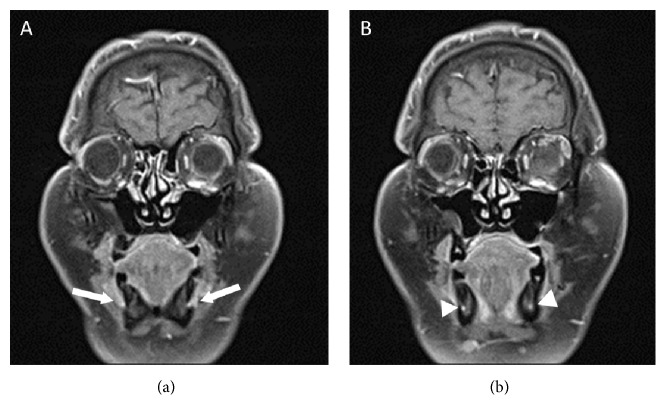
Serial coronal contrast-enhanced T1-weighted images of the face showing perineural infiltration with thickening and enhancement of the mental nerves bilaterally (arrows) with involvement of the inferior alveolar nerves as well (arrowheads).

A 39-year-old male patient, newly diagnosed with diabetes mellitus type 2 and hypertension, presented with bilateral numbness in the lower lip and chin for two-week duration. The numbness was continuous with no associated pain, weakness, or any other neurological symptoms. There was no history of recent trauma or dental extraction. Three weeks later, he presented with bilateral lower limb erythema and swelling and was admitted as a case of cellulitis for treatment. He also complained of bony pain especially in the back and hips, and he lost more than 30 kg in three months. There was no history of fever, fatigue, headache, or lymph node enlargement. On examination, the patient was febrile with a temperature of 37.7°C and a heart rate of 112 beats per minute with normal blood pressure and oxygen saturation. He was morbidly obese with excessive sweating, but there was no pallor or jaundice. No lymphadenopathy was clinically detectable. Abdominal examination revealed left hypochondrial mass measuring around 15 × 10 cm, which was firm and tender. Neurological examination was normal apart from hearing loss, hypoesthesia to thermal stimulation, and pinprick over the distribution of mental nerve bilaterally. Lower limb examination showed bilateral lower limb erythematous rash with multiple dark lesions. Investigations revealed normal WBC count and hemoglobin but low platelets level (36,000). Lactate dehydrogenase and uric acid were high, but the rest of his biochemical profile was normal. Flow cytometry of peripheral blood showed 8% blast cells and was positive for CD9, CD10, CD19, CD20, CD38, and HLADR, and it was negative for CD4, CD11b, CD13, CD15, CD33, CD34, CD64, CD117, CD123, and surface immunoglobulin kappa and lambda light chain. Bone marrow examination showed 80% blast cells with a high nuclear : cytoplasmic (N : C) ratio, basophilic cytoplasm, irregular nuclear membrane, and some vacuolation, which was consistent with acute leukemia by morphology. Flow cytometry of bone marrow aspirate showed 55% blast cells consistent with precursor B-cell acute lymphocytic leukemia. Cytogenetic analysis showed translocation 8 : 14, which is consistent with Burkitt's lymphoma. Lower limb skin biopsy showed percutaneous leukemic infiltrate cells consistent with precursor B-cell acute lymphocytic leukemia. MRI of the brain was normal, but MRI of the face showed bilateral abnormal enhancement of the mental nerve at the level of the mental foramen, most likely related to leukemic infiltration (Figure 5). CFS analysis showed a clear fluid with no WBCs, RBCs, or blast cells and normal biochemical profile. The final diagnosis was Burkitt's lymphoma/leukemia. He was started on dexamethasone, allopurinol, and chemotherapy (hyper-CVAD protocol with intrathecal chemotherapy). He was also given rituximab, methotrexate, and cytarabine. The patient developed febrile neutropenia with positive culture for pseudomonas and* Acinetobacter* with small perianal abscess for which he was treated aggressively and successfully. He finished his full regimen of chemotherapy cycles with complete resolution of his symptoms and signs and normalizations of his laboratory parameters.

## 4. Discussion

The mental nerve is a branch of the inferior alveolar nerve which is the posterior trunk of the mandibular division of the trigeminal nerve. The mental nerve divides into three branches: one descends to the skin of the chin, and two ascend to the skin and mucous membrane of the lower lip. The function of the mental nerve is purely sensory innervating the lower lip and chin and the lower gingiva and teeth [4].

Neuropathy of the mental nerve and its branches are attributed to either benign or malignant conditions. The most common causes are odontogenic such as local trauma, dental abscesses, anesthesia, osteomyelitis, and mandibular cysts. Mechanical compression of the nerve or malignant infiltration along the nerve sheath is common causes of this syndrome related to several malignancies [5].

Numb chin syndrome was first described by Charles Bell in 1830 as facial numbness localized to the distribution of the mental nerve and its branches and was named numb chin syndrome [6]. Mental neuropathy associated with malignancy was first reported in 1963 by Calverley and Mohnac [7] who described five patients with metastatic malignant disease who had numb chin syndrome as the initial presentation. Malignancies associated with this syndrome could be solid or hematologic. Solid malignancies include thyroid, prostate, lung, and breast cancer. Hematologic malignancies include multiple myeloma, acute lymphocytic leukemia, and lymphoproliferative processes such as lymphoma. Numb chin syndrome usually appears within the course of known cancer but rarely precedes the diagnosis and is the presenting symptom, as in our cases [8]. However, it usually precedes the diagnosis and is the first manifestation in hematologic malignancies in general and Burkitt's lymphoma in particular [2]. It was also reported in association with systemic diseases such as sarcoidosis, amyloidosis, HIV, syphilis, diabetes mellitus, multiple sclerosis, and sickle cell disease [8].

Diagnosis of numb chin syndrome is mainly clinical, but imaging and laboratory studies are required to confirm the diagnosis and show the area of nerve involvement and the etiology. Although panoramic radiography of the jaw is a useful starting point, most practicing physicians start with either CT or MRI of the jaw, face, and brain. Radiographic findings may include osteoblastic or osteolytic lesion anywhere along the mandible or in the region of mental foramen. In addition, underlying dental pathology such as thinning of lamina dura or tooth displacement have also been discovered in more than 50% of patients with underlying hematologic malignancy [9]. CT imaging of the brain and skull base may reveal evidence of brain metastasis or leptomeningeal invasion. CT imaging of the mandible performed in several planes can yield a clear and accurate image of the inferior alveolar nerve and its surroundings. CT scan is more sensitive than panoramic radiography. MRI of the face and the brain is extremely useful for evaluating numb chin syndrome. Some authors recommend MRI scanning of the face if the etiology of mental neuropathy is unclear after routine imaging studies have been done. Nuclear bone scanning may identify mandibular bone diseases such as metastasis and osteomyelitis and metastasis elsewhere in the skeleton. Lumbar puncture with cytological analysis of the CSF may also be necessary to exclude leptomeningeal metastasis or carcinomatous meningitis if imaging studies fail to reveal anatomical lesion [9]. In one study, the combination of cytological analysis of the CSF in conjunction with CT scan of the mandible, base of the skull, and brain yielded the diagnosis in 89% of patients with numb chin syndrome and known malignancy [10]. Fan et al. reported a case of numb chin syndrome where the CSF findings revealed no malignant cells and imaging findings did not reveal evidence of metastasis in brain parenchyma, CSF, or mandibular bone. The MRI abnormalities were diffuse dural thickening and focal lesion in clivus. The authors suggested a possible mechanism of microscopic seeding of the malignant cell in the nerve, which is not visible in radiological imaging. This could be another mechanism causing numb chin syndrome [11].

The treatment of numb chin syndrome is targeted towards the underlying disease especially in cases of dental diseases such as dental abscesses. In patients with numb chin syndrome caused by metastatic cancer, treatment should target the underlying malignancy. Local and cranial irradiation have been utilized in patients with skull base metastasis and leptomeningeal metastasis, respectively. Mandibular lesions do not typically require local radiotherapy because symptoms of the syndrome resolve spontaneously. The prognosis is poor and most patients survive less than a year [12].

Burkitt's lymphoma is a highly aggressive germinal center B-cell-derived non-Hodgkin lymphoma. It is the fastest growing human cancer and the most common childhood cancer in Africa where malaria is endemic, and it has a high incidence in immunocompromised hosts especially HIV patients. It is associated with Epstein-Barr virus (EBV) and has a chromosomal translocation that activates MYC oncogene. The prognosis in children has improved but is poor in older adults. The world health organization classified Burkitt's lymphoma into three clinical variants: endemic (in malaria-endemic areas and associated with EBV), sporadic (in the rest of the world and rarely associated with EBV), and immunodeficiency-related (in HIV patients and EBV is present in less than 40% of the cases) [13]. The most common site of presentation of the sporadic form is the abdomen (as seen in our patients) followed by the head and neck region. The bone marrow is infiltrated in about 20% of patients, and they are classified as Burkitt's lymphoma/leukemia if the infiltration is extensive, as in our cases [14].

Diagnosis of Burkitt's lymphoma is confirmed by microscopy, immunocytological analysis, cytogenetics, and molecular studies. The hallmark of Burkitt's lymphoma is the genetic translocation t(8;14)(q24;q32), and it is present in 70–80% of the affected patients [15]. Treatment of Burkitt's lymphoma is guided by clinical and histopathological staging. Prognosis depends on staging which includes the disease extent. The treatment outcome of sporadic cases of Burkitt's lymphoma in high-income countries is excellent with about 90% cure rate. However, the outcome in adult patients, especially the elderly, is poor since they present with rapidly progressive disease. Relapse of Burkitt's lymphoma is associated with poor prognosis, possibly because the strongest treatment regimens have been already exhausted and few drug choices remain [16].

The association between Burkitt's lymphoma and numb chin syndrome has been documented in the literature with bilateral association being the most common. Sasaki et al. [2] reviewed the literature and found bilateral numb chin syndrome associated with malignancy in 22 cases. They found that bilateral presentation was associated with hematologic malignancies more than with solid malignancies. This could be explained by the fact that hematologic malignancies infiltrate the central nervous system more readily than the solid malignancies. The syndrome preceded the diagnosis in 8 cases and was associated with progression, recurrence, or metastasis of the primary tumor in the remaining 14 patients. It is important to note that, in three of these cases in which the syndrome preceded the diagnosis, the initial blood tests were normal, but later blood tests revealed a hematologic malignancy, as in our first case. This illustrates the importance of repeating the blood tests in these patients and not excluding malignancy from the differential diagnosis until a definite cause has been established [2]. This is in contrast with the fact that numb chin syndrome usually occurs in the setting of a known cancer or is a sign of recurrence or metastasis, which illustrates the importance of considering the initial bilateral numb chin syndrome as a red flag and raise the suspicion for Burkitt's lymphoma particularly or other underlying malignancy in general [8].

## 5. Conclusion

In conclusion, numb chin syndrome should be considered as a warning sign and raises the suspicion for an underlying malignancy. Bilateral involvement is especially hazardous and must not be underestimated. In fact, an astute neurologist and internist who realizes that chin numbness is a potentially significant symptom can then exclude serious underlying malignancies. Standard diagnostic protocol with different modalities of imaging based on the availability and experience of the radiology team should be mandatory. High index of suspicion should be practiced to avoid delay in diagnosis and progression of the underlying malignancy.
